# Feasibility, safety, and efficacy of high-dose intermittent theta burst stimulation in children with autism spectrum disorder: study protocol for a pilot randomized sham-controlled trial

**DOI:** 10.3389/fpsyt.2025.1549982

**Published:** 2025-03-31

**Authors:** Junzi Long, Maoyuan Niu, Xingxing Liao, Kaiyue Han, Jiarou Chen, Wenlong Su, Xianna Wang, Jianjun Liu, Yan Zhang, Hao Zhang

**Affiliations:** ^1^ School of Rehabilitation, Capital Medical University, Beijing, China; ^2^ Department of Neurorehabilitation, Beijing Boai Hospital, China Rehabilitation Research Center, Beijing, China; ^3^ Division of Brain Sciences, Changping Laboratory, Beijing, China; ^4^ China Autism Rehabilitation Research Center, Beijing Boai Hospital, China Rehabilitation Research Center, Beijing, China; ^5^ The Second Affiliated Hospital and Yuying Children’s Hospital, Wenzhou Medical University, Wenzhou, Zhejiang, China; ^6^ SanBo Brain Hospital, Capital Medical University, Beijing, China

**Keywords:** autism spectrum disorder, theta burst stimulation, repetitive transcranial magnetic stimulation, feasibility, safety, efficacy, study protocol

## Abstract

**Background:**

Autism spectrum disorders (ASD) are common neurodevelopmental disorders, mainly caused by disrupted excitation/inhibition balance and synaptic plasticity. Intermittent theta burst stimulation (iTBS) is a variant of excitatory repetitive transcranial magnetic stimulation, inducing long-term potentiation-like plasticity. In recent years, there has been a growing interest in high-dose iTBS as a therapeutic tool for psychiatric disorders. We aim to preliminarily investigate the feasibility, safety, and efficacy of high-dose iTBS in children with autism spectrum disorder (ASD).

**Methods:**

A randomized controlled pilot trial with a 4-week intervention will be conducted. Forty children with ASD will be randomized into either the intervention or control group. The intervention group will receive 5400-pulse iTBS per day, while the control group will receive sham iTBS. Feasibility will be evaluated through recruitment, intervention adherence, and assessment completion. Safety will be assessed by comparing the rates of drop-outs attributed to adverse events and the rates of serious adverse events The efficacy outcomes include the Autism Behavior Checklist, Social Responsiveness Scale, 2nd Edition, Childhood Autism Rating Scale, Autism Treatment Evaluation Checklist and Repetitive Behavior Scale-Revised. Resting-state electroencephalogram and functional near-infrared spectroscopy will be employed to quantify alterations in functional brain connectivity and cerebral haemodynamics. Salivary levels of oxytocin, growth hormone, insulin-like growth factor 1, and insulin-like growth factor binding protein 3 are measured to reflect the biochemical response to iTBS. These indicators will be assessed at baseline and at the end of the intervention.

**Discussion:**

This trial will evaluate the feasibility, safety, and efficacy of high-dose iTBS treatment in children with ASD. The proposed study will provide pilot data to inform the feasibility and design of larger sample-size trials.

**Clinical trial registration:**

http://www.chictr.org.cn, identifier ChiCTR2400089757.

## Introduction

Autism spectrum disorder (ASD) is a neurodevelopmental disorder that manifests in early childhood, with an estimated global prevalence of 0.72% (1). Children with ASD exhibit persistent deficits in social communication and restricted, repetitive patterns of behavior or interests (2). Treatments for young children with ASD are primarily behavioral interventions but can be a significant burden on families in terms of time and resources. Although medications (e.g. risperidone and aripiprazole) are evidenced to manage the accompanying symptoms of ASD, there remains a lack of effective treatment for the core symptoms of ASD (3, 4). Hence, more studies are urgently needed to elucidate the pathogenesis of ASD and explore potential effective treatment options.

Among the potential pathophysiological mechanisms implicated in ASD is the excitation/inhibition imbalance. Repetitive transcranial magnetic stimulation (rTMS) can induce a rapidly changing magnetic field that generates an electrical current in the target brain region, leading to both inhibitory or excitatory neuronal changes. Over the recent years, rTMS has been proposed as a neurostimulation technique with evidence-based efficacy in treating ASD (5, 6). Theta burst stimulation (TBS) is a modified variant of rTMS that simulates the frequency of the hippocampal theta rhythm. TBS comprises two main types: intermittent TBS (iTBS), which induces long-term potentiation-like effects, and continuous TBS (cTBS), which induces long-term depression-like effects (7, 8). The preceding neuroscience-informed consensus and our meta-analysis indicate that the DLPFC may represent a promising stimulation target for ASD (9, 10). Applying rTMS over the left dorsolateral prefrontal cortex (DLPFC) has been demonstrated to significantly improve various symptom domains in neuropsychiatric disorders, including craving, depression, anxiety, obsessions and compulsions, pain perception, global cognition, declarative memory, working memory, cognitive control, and motor coordination (11). Furthermore, the left DLPFC was most frequently targeted in the studies using TMS to treat ASD (5). Compared to the right DLPFC, iTBS over the left DLPFC exerts a more pronounced modulatory effect on various cognitive domains. cTBS over the left DLPFC impairs executive function, working memory, auditory feedback regulation, and cognitive control, while cTBS over the right DLPFC impairs inhibitory control, planning, and attention (12). In children with ASD, cTBS over the left DLPFC has been shown to have no significant impact on autistic symptoms and white matter macro/microstructure and connection (13, 14). Conversely, 30 sessions of iTBS treatment over the left DLPFC were found to alleviate depressive symptoms in individuals with ASD (15). An interventional course of excitatory rTMS (20 Hz) targeting the left DLPFC can modulate the glutamatergic system in individuals with ASD (16). It can be reasonably proposed that iTBS interventions targeting the left DLPFC may represent a promising rTMS treatment strategy for the management of autistic symptoms. However, the currently available evidence base is largely comprised of case series with overall low-quality evidence, and there is a notable absence of randomized controlled trials.

In recent years, the use of high-dose iTBS has emerged as a promising rTMS approach for psychiatric and neurological disorders. For example, researchers discovered that both a prolonged iTBS (1800 pulses/day, 10 days) and an accelerated high-dose iTBS intervention (1800 pulses/session, 10 sessions/day, 5 consecutive days) over the left DLPFC were efficacious in ameliorating the severity of refractory depression, and all subjects exhibited tolerability to the treatments without significant adverse events (17, 18). Accelerated high-dose iTBS targeting the left DLPFC was also shown to improve cognitive dysfunction in patients with Alzheimer’s disease, which may be related to its enhancement of cortical excitability and β-oscillatory activity (19). Our recent researches have demonstrated that high-dose iTBS has the potential to improve upper limb motor function and cognitive function in stroke patients with no significant safety concerns (20, 21). It is noteworthy that the majority of iTBS trials for ASD have employed conservative dosages, with a daily dosage of 600 pulses (15, 22, 23). A preliminary MRI analysis revealed that four weeks of iTBS intervention targeting the bilateral posterior superior temporal sulci in children with ASD did not result in significant improvements in social communication or reductions in repetitive behaviors. Additionally, no notable changes were observed in the macroscopic or microstructural properties of cerebral white matter. These findings may be attributed to an insufficient stimulation dosage (1200 pulses per hemisphere per session, one session per day, twice a week for four weeks) (24). As indicated by Smith et al. in a recent systematic review, relative to current TMS research on other neuropsychiatric diseases, the number of TMS pulses administered in ASD participants was significantly lower, raising concerns about underdosing (5). A recent open-label trial was the first to investigate the effects of a higher dose of high-frequency rTMS (3000 pulses per day for 25 days) on the left DLPFC in adults with co-morbid ASD and major depressive disorder. The results indicated the rTMS treatment was well-tolerated by participants, with improvement in depressive symptoms and possible effects on core autism symptoms (25). Moreover, the improvement of rTMS on autistic symptoms also presents a dose-response relationship (26). Recently, a growing number of researchers have advocated for the optimization of TMS treatment protocols for ASD, specifically exploring varied stimulation dosages to uncover additional clinical benefits (27, 28). To sum up, future studies investigating the feasibility, safety, and efficacy of high-dose rTMS treatment in ASD are necessary.

A recent systematic review suggests that the reported adverse events in children undergoing TBS interventions were mild and similar to those noted in adult studies, suggesting comparable safety, tolerability, and feasibility between the two groups (29). According to a recent meta-analysis, all reported adverse events of rTMS in ASD are mild and transient, with an overall prevalence of 25% (30). Similarly, a meta-analysis indicates that both rTMS and TBS can be safe therapeutic options for intellectually capable pediatric and young adult individuals with ASD (5). Prior adolescent works suggest that high-frequency rTMS to the left DLPFC is feasible, safe, and effective for major depressive disorder over the course of 30 sessions (3,000 pulses per session, 90000 pulses in total) (31, 32). In another study, employing 10 Hz rTMS treatment with 2500 pulses of 40 sessions (100000 pulses in total) over the primary motor cortex significantly decreased muscle spasticity in cerebral palsy children without any adverse effects or seizures (33). Compared to the adult literature, however, data on the safety of high-dose rTMS in pediatric populations are relatively lacking. Considering the promising role of high-dose rTMS treatment in adult neuropsychiatric disorders, future dose-ranging studies with assessment of adverse events will be important in translating the results of rTMS from adults to pediatric populations (29). In sum, the aforementioned findings provide us insights into the preliminary investigation of the feasibility, safety, and efficacy of high-dose iTBS protocol (5400 pulses/day, 5 days/week, 4 weeks, 108000 pulses in total) in children with ASD.

Resting-state Electroencephalogram (EEG) can reveal intrinsically connected functional networks, with increased applications in the therapeutic effect evaluation of rTMS on ASD. For example, prior research has demonstrated the hyper-variability in the resting-state networks of individuals with ASD, and a three-week course of rTMS alleviated the hyper fluctuations in the frontal-parietal and frontal-occipital connectivity, which in turn contributes to the amelioration of ASD symptoms (34). By employing resting-state EEG, Kang et al. (35) discovered that following 18 sessions of low-frequency rTMS on the DLPFC region in children with ASD, the effective connectivity in the alpha band diminished from O1 to T7, and in the gamma band from Pz to T8. Functional near-infrared spectroscopy (fNIRS) can be used to reflect changes in cerebral blood flow thus offering a visual representation of brain functional activities, which has been widely utilized in the researches of ASD. A fNIRS study based on an expression recognition task found that after treatment with low-frequency rTMS, oxyhemoglobin levels in the left DLPFC decreased and those in the right DLPFC increased when children with ASD recognized happy expressions, suggesting that rTMS may affect neural functional activity during expression recognition (36). In sum, the application of EEG and fNIRS techniques enables a more profound comprehension of the alterations in cerebral hemodynamics and functional brain connectivity attributes in children with ASD, both prior to and following rTMS therapy.

A number of studies have highlighted the regulatory effect of the excitatory form of rTMS on the hypothalamic-pituitary-adrenal/thyroid axis by targeting the left DLPFC, including the regulation of levels of cortisol, thyroid-stimulating hormone, and free thyroxine (37, 38). Oxytocin is hypothalamic-posterior pituitary hormone with prosocial effects and its level is lower in autistic children (39). A six-week course of excitatory rTMS (10Hz) over the left DLPFC has been observed to increase salivary oxytocin levels in patients with depression who exhibit low basal oxytocin levels (40). The growth hormone is a pivotal polypeptide hormone that is secreted by the anterior pituitary gland, and its secretion is subject to regulation by hypothalamic neuropeptides. Insulin-like growth factor 1 (IGF-1) is mainly stimulated by growth hormone and has growth promoting effects in various body cells. The major functions of insulin-like growth factor binding protein 3 (IGFBP-3) are to facilitate the transport of IGF-1, prolong its half-lives, and regulate its interactions with IGF receptors (41). Children with ASD have significantly higher levels of several growth hormone-related factors, including growth hormone, IGF-1, and IGFBP-3, which could be related to their significantly larger head circumferences and higher weights than neurotypical controls (42, 43). It is currently known that activation of the left DLPFC induced by high-frequency rTMS may exert a top-down control of the hypothalamic-pituitary-adrenal axis leading to decreased cortisol secretions (44). A study of 15 subjects with major depressive disorder revealed that ten sessions of 1-Hz rTMS treatment targeting the right-sided DLPFC resulted in a reduction in serum growth hormone levels in the patients (45). Moreover, 1-Hz rTMS applied to the left DLPFC resulted in a trend toward decreased serum IGF-1 levels in autistic subjects from pre- to post-treatment (46). However, the regulatory effect of the excitatory form of rTMS over the DLPFC on the growth hormone-IGF-1 axis remains unclear. The above findings suggest that rTMS may indirectly influence the hypothalamic-pituitary axis via the cerebral cortex (e.g., DLPFC), potentially regulating the levels of oxytocin and growth hormone-related factors. This mechanism may serve as a pathway through which rTMS exerts its action in improving the core symptoms of ASD, a hypothesis that requires further investigation.

In light of these evidences, we designed a pilot randomized controlled clinical trial to investigate the feasibility, safety, and efficacy of high-dose iTBS in children with ASD. We hypothesize that high-dose iTBS may be a feasible and safe rTMS protocol for children with ASD. A 4-week high-dose iTBS intervention, compared to sham stimulation, is expected to result in significant improvements in core autistic symptoms, alterations in resting-state functional connectivity, and changes in salivary levels of oxytocin, growth hormone, IGF-1, and IGFBP-3.

## Methods and analysis

This trial was registered on the China Clinical Trial Center (ChiCTR2400089757). This manuscript was written in accordance with the Standard Protocol Items: Recommendations for Interventional Trials guidelines (47). The study was reviewed and approved by the Ethical Committee of the China Rehabilitation Research Center, under the number: 2024-085-02.

### Study design and setting

**Figure 1:**
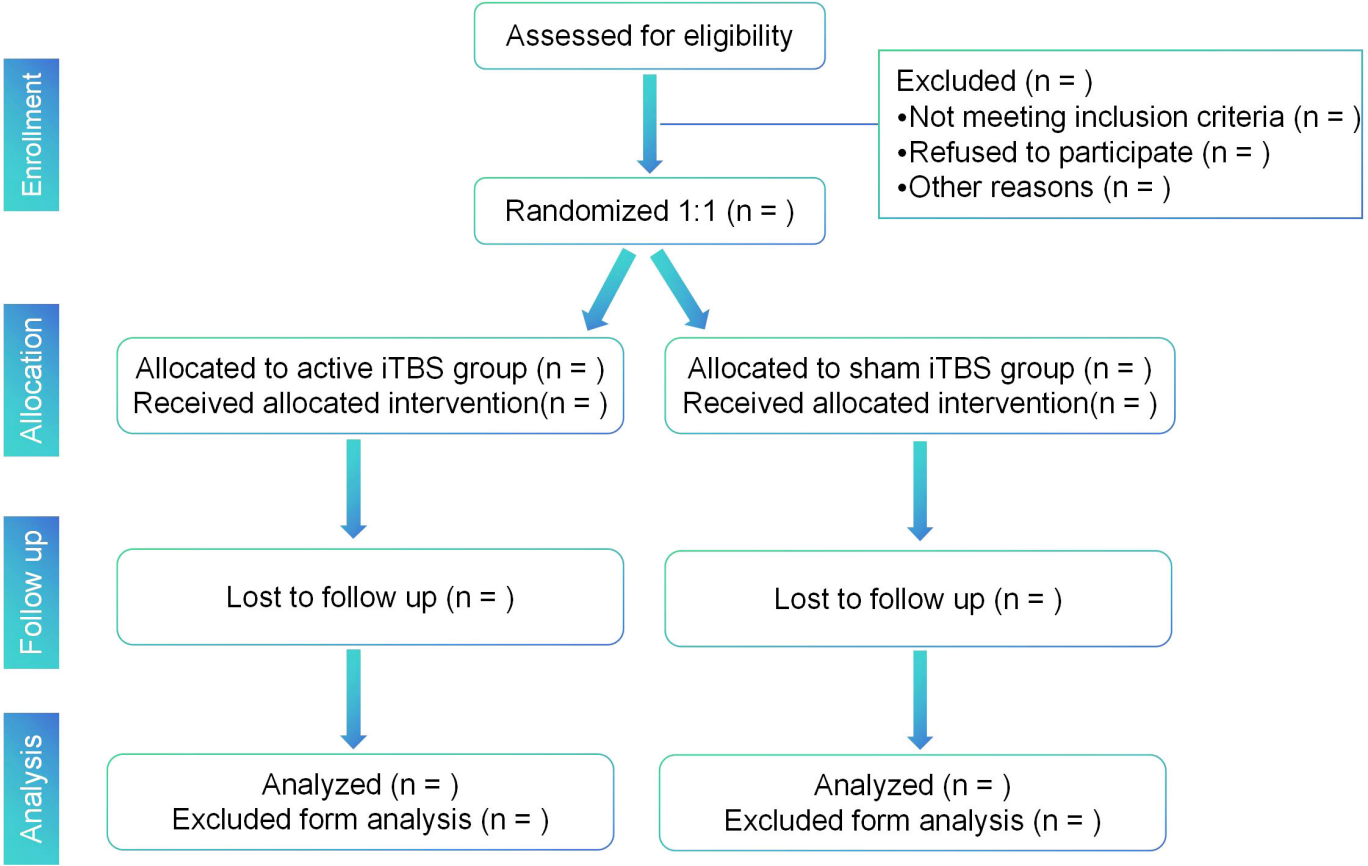
Study flow diagram. iTBS, intermittent theta burst stimulation.

**Figure 2:**
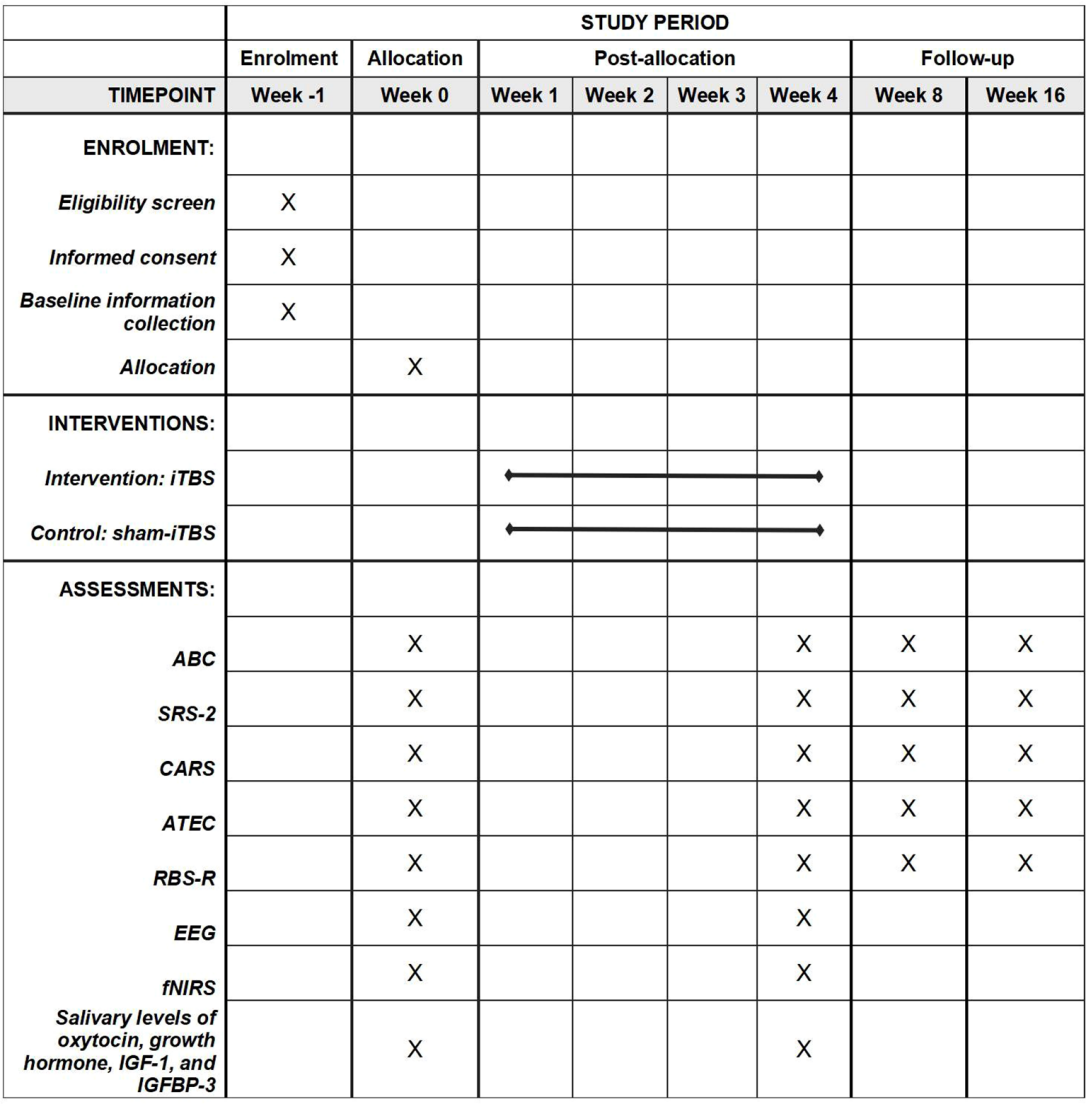
The schedule of enrolment, interventions and assessments. ABC, Autism Behavior Checklist, ATEC, Autism Treatment Evaluation Checklist; CARS, Childhood Autism Rating Scale; EEG, electroencephalogram; fNIRS, functional near-infrared spectroscopy; IGF-1, insulin-like growth factor-1; IGFBP-3, insulin-like growth factor binding protein-3; iTBS, intermittent theta burst stimulation; RBS-R, Repetitive Behavior Scale-Revised; SRS-2, Social Responsiveness Scale, 2nd Edition.

This study is a two-arm, double-blind, placebo-controlled, pilot randomized controlled trial involving children with a diagnosis of ASD. We will explore the feasibility, safety, and efficacy of high-dose iTBS in children with ASD. Subjects will be randomly assigned to either the active or sham group for a four-week course of high-dose iTBS administration. The study will be conducted in a room specifically designated for rTMS procedures at the Beijing Boai Hospital (a Grade A tertiary hospital) in Beijing, China. All members of the study will adhere to the principles of Good Clinical Practice and the Declaration of Helsinki. The study will be conducted from September 2024 to June 2026. The flowchart of the study protocol is depicted in **Figure 1**. The schedule of enrollment, interventions, and assessments is shown in **Figure 2**.

### Eligibility criteria

Subjects will be recruited from the China Autism Rehabilitation Research Center, Beijing Boai Hospital, if they are aged between 2–10 years, with an ASD diagnosis according to the DSM-5 criteria. The majority of individuals with ASD who come to our hospital for treatment and hospitalization are between 2 and 10 years old, which ensures the feasibility of including children of this age group. We include pre-adolescent children to reduce the influence of pubertal development on the outcomes (e.g. biological outcomes). The statement of informed consent should be signed by a legal guardian. Both gender and education level are not limited. Exclusion criteria are as follows: 1) History of seizures or epilepsy; 2) Neurological or psychiatric disorders not associated with ASD; 3) Cardiac pacemakers, cochlear implant, and any metal inserted into the head; 4) Having undergone some neurosurgical procedures; 5) Currently participating in another randomized controlled trial; 6) Receiving non-invasive brain stimulation in the past 3 months; 7) Concomitant psychopharmacological medication or medication contraindicated for rTMS. Participants who are absent from two or more consecutive intervention sessions or a total of four sessions will be withdrawn from the study. Subjects will also be withdrawn from the study if they are unable to cooperate with the treatment or if their legal guardian is unwilling to continue with the trial. The study of a participant will end if he/she has a serious adverse reaction to the treatment, such as an epileptic seizure.

### Intervention procedures

Twenty intervention sessions will be held over 4 consecutive weeks, with five sessions per week (Monday to Friday). A repetitive magnetic stimulator (Neurosoft LLC, 5, Voronin str., Ivanovo, 153032, Russia) and a figure-of-eight coil with a 70 mm diameter will be employed to administer iTBS over the left DLPFC (F3), following the 10–20 international EEG system (48). The subjects are required to wear a tight-fitting EEG cap, and the position of “F3” is marked using a mark pen. Three different sizes of EEG caps will be prepared for the purpose of target locating assistance, which will correspond to the following head circumferences: 46-50 cm (extra small), 50-54 cm (small), and 54-58 cm (medium). The magnetic coil was positioned at this point with an angle of approximately 45° to the interhemispheric line (49). A “sham coil” is employed to simulate the visual, auditory, and tactile aspects of rTMS without actually delivering any electromagnetic stimulation. The resting motor threshold of each participant was determined by stimulating the primary motor cortex until a 50 μV deflection of electromyogram or a visible twitch in the first dorsal interosseous muscle was identified in at least two of three trials (26, 50). One iTBS session consists of triplet 50 Hz pulses repeated in 5 Hz bursts for a total of 1800 pulses at 90% of the motor threshold (51). Sixty trains were applied in 2-second bursts with 8-second pauses. Previous research has found that three continuous sessions of iTBS with no interval (1800 pulses) over the primary motor cortex can reverse the effect from excitatory to inhibitory (52). Contrary to this motor cortex study, applying iTBS with 1800 pulses over the left DLPFC may act on DLPFC activity via the modulation of excitation/inhibition balance, which is similar to one regular session of iTBS (600 pulses) (53). The treatment will be administered in three sessions per day, with a 10-minute interval between each session (5400 pulses per day). The magnetic coil will be held by the experimenter as this is more comfortable for the subjects. During the rTMS administration, the subjects will be instructed to sit in a chair and refrain from moving. Children who are emotionally unstable or unable to remain seated are allowed to eat snacks, play with their favorite toys, or listen to music, while parents are encouraged to offer comfort and reassurance. Earplugs are provided for noise reduction. Younger children can be held by their parents or placed in a stroller for treatment. If adaptation remains challenging, parents are advised to bring their child to the treatment room daily over the next few days to acclimate them to the room environment and the noise and stimulation of TMS. For children who continue to be unable to tolerate treatment, they will be withdrawn from the trial. Once the child has acclimated to TMS treatment, eating snacks, playing with toys, or listening to music during the sessions will no longer be allowed. The children are allowed to rest between the three treatment sessions, during which they are free to play and interact with the parents and researchers. Furthermore, it is our intention to cultivate a positive relationship with the children.

### Assignment of interventions

This is a double-blind randomized clinical trial. The randomization of participants will be conducted using a computer-generated random list, based on the permutation block method with a random number generator. The ratio of participants allocated to each group will be 1:1. The allocation sequence was concealed by a research assistant using sealed, opaque envelopes that contained the treatment information prior to the study’s official commencement. Once the consent form and baseline measures have been completed, an envelope for a participant ID number will be provided to the rTMS therapist prior to the first iTBS session. The rTMS therapist is the sole individual responsible for determining the treatment method (iTBS or sham-iTBS), and he/she does not perform assessments or statistical analyses. It is imperative that other study researchers, participants, parents/guardians, and statistical analysts, remain completely blinded to the treatment condition. Any adverse or discomfort event will be recorded after each iTBS treatment session. If a participant experiences a serious adverse event, the blindness will be removed.

### Sample size and recruitment

Forty children diagnosed with ASD will be included in total (iTBS group, n = 20; sham group, n = 20). Whilst a formal sample size calculation is not required in a pilot study (54), it is recommended that a sample size of between 15 and 20 participants is maintained for the purpose of conducting a subsequent full-scale study (55). In consideration of a potential dropout rate of 15%, the objective was to recruit a total of 48 participants. Nonetheless, we will endeavor to recruit a greater number of participants if feasible within the time and resources available. All participants will be recruited from the China Autism Rehabilitation Research Center, Beijing Boai Hospital, where advertisements will be posted to attract potential participants. One researcher will conduct the recruitment process, during which he/she will document the participants’ medical histories, including past and current medical conditions.

### Outcome measures

For the first study objective, to determine feasibility and safety, we will report the recruitment rate, adherence rate, completion rate, and adverse reactions. The recruitment rate will be reported as the percentage of eligible ASD children who will participate in the study and the number of subjects recruited monthly over the recruitment period. The adherence rate will be measured by the proportion of participants who complete the intervention as planned. The completion rate will be assessed by the proportion of participants who complete all assessments and follow-ups. Safety will be assessed by comparing the rates of drop-outs attributed to adverse events and the rates of serious adverse events. For the second study objective, to determine the potential efficacy of the iTBS intervention on ASD, our endpoints include the Autism Behavior Checklist (ABC), Social Responsiveness Scale, 2nd Edition (SRS-2), Childhood Autism Rating Scale (CARS), Autism Treatment Evaluation Checklist (ATEC) and the Repetitive Behavior Scale-Revised (RBS-R). The ABC scale consists of 57 items that involve a range of typical autistic behaviors. These items are categorized into five domains including relating, sensory, language, body use and object manipulation, and social and self-help. Each item contained its own score ranging from 1 to 4 points according to the item weights, and higher total scores indicate more severe ASD symptoms (56) The SRS-2 is a norm-referenced quantitative assessment comprising 65 items pertaining to children’s behavioral characteristics. Parents or caregivers are required to rate five subdomains, namely social awareness, social cognition, social communication, social motivation, and restricted repetitive behaviors (57). The CARS was developed as an observational rating scale to capture ASD symptoms through parent/carer interviews, observations, and case reviews. Each item is scored from 1 to 4, with 1 representing appropriate behavior and 4 representing behavior that deviates severely from normal criteria, so higher total scores indicate more severe ASD symptoms. This tool can be utilized to display the overall symptom severity of ASD (58). The participant’s parent needs to complete the ATEC questionnaire, which is comprised of four subscales (speech/language communication, sociability, sensory/cognitive awareness, and physical/health/behavior). The ATEC has been used to measure treatment effects, with a lower score indicating a lower severity of ASD symptoms (59). The RBS-R was a valid tool for assessing repetitive and stereotypic behaviors in children with ASD, including six parts: stereotyped behaviors (6 items), self-injurious behaviors (8 items), obsessive-compulsive behaviors (8 items), ritualistic behaviors (6 items), fixation behaviors (11 items), and restrictive behaviors (4 items). The scale uses a 0-3 scoring system: 0 for no behavioral problems, 3 for severe, 2 for moderate, and 1 for mild (60).

Salivary levels of oxytocin, growth hormone, IGF-1, and IGFBP-3 are measured by ELISA to reflect the biochemical response to treatments. A number of studies have demonstrated that the ELISA is capable of testing the concentration of oxytocin, growth hormone, IGF-1, and IGFBP-3 in human saliva (61–63). The ELISA kits are purchased from Shanghai Enzyme-linked Biotechnology Co., Ltd. (Shanghai, China). The parents/legal guardians were requested to ensure that their children refrained from eating, drinking, and oral cleaning for at least an hour before saliva collection. Subjects were instructed to spit at least 0.5ml of saliva into a disposable saliva collector. If a child is unable to carry out this instruction, the researchers will use a disposable pipette to extract his/her saliva. To eliminate any potential bias due to circadian rhythms, all salivary samples were collected between 4 and 5 p.m. Each sample was centrifuged and the resulting supernatant was divided into Eppendorf tubes and frozen at -80°C. At the end of the trial, the saliva samples will be irrevocably destroyed.

Resting-state EEGs will be recorded for 10 minutes using the CURRY 8.0 software in a 64-channel Neuroscan system (Compumedics Neuroscan, Victoria, Australia). An EEG cap with saline electrodes is used (GREENTEK, Wuhan, China) based on the international 10-20 system, which is easy to apply and does not necessitate gel applications. The data are acquired at a sampling rate of 1 kHz, and the impedances are maintained at a level below 50 kΩ. All the children need to be awake and relaxed during the EEG recordings. Three types of caps (extra small, small, and medium) will be prepared for them.

**Figure 3:**
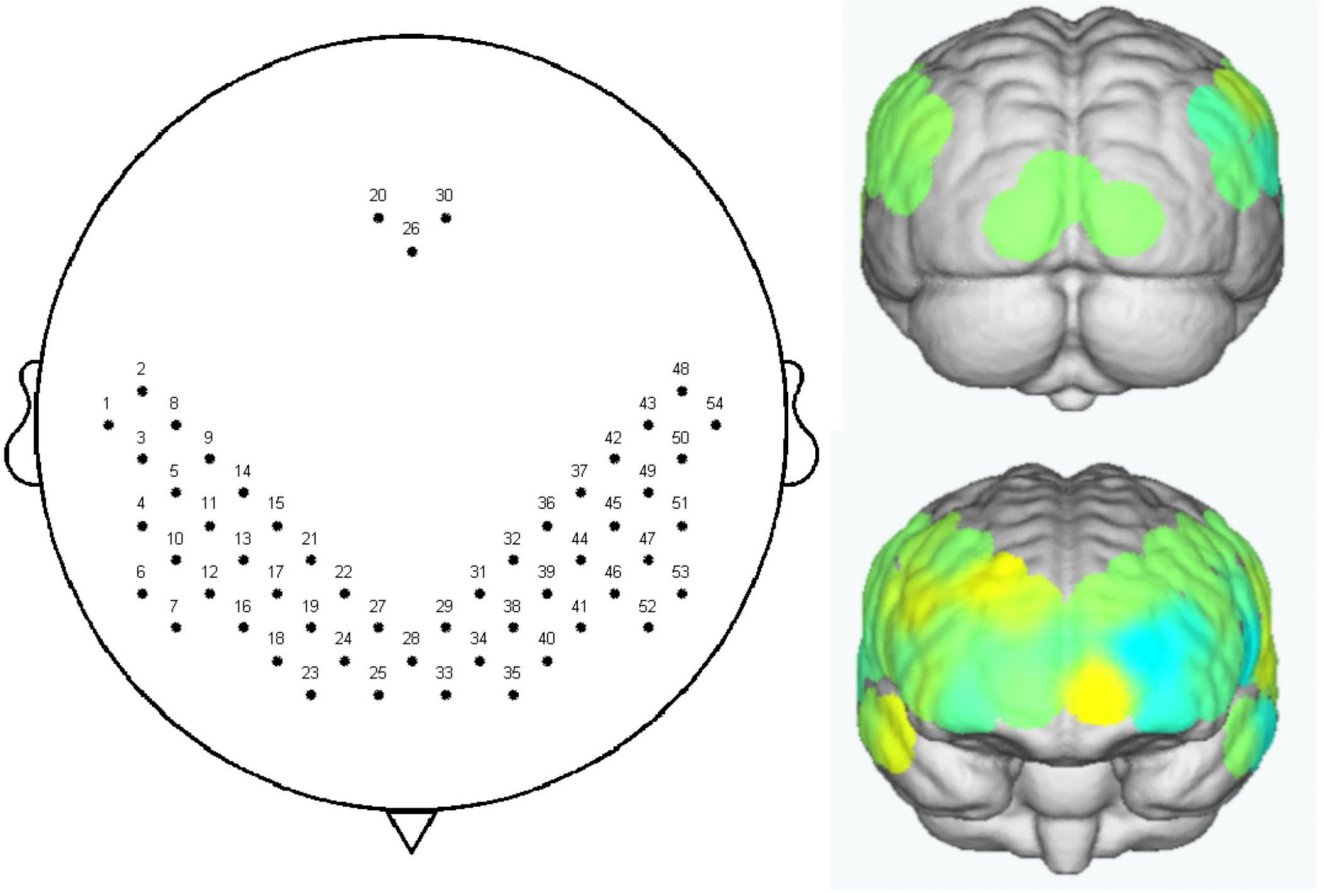
The channel layout and area of interest in functional near-infrared spectroscopy.

A fNIRS system (NirSmart-6000A, Danyang HuiChuang Medical Equipment Co., Ltd., Zhenjiang, China) is used to measure the spontaneous fluctuations in hemoglobin concentration in children with ASD at the resting state. The absorptions of three wavelengths (780nm, 805nm, and 830nm) of near-infrared light are measured with a sampling rate of 14.286 Hz (i.e. 70-ms temporal resolution) and subsequently transformed into concentration changes of oxyhemoglobin, deoxyhemoglobin, and total hemoglobin by means of the modified Beer-Lambert law. Fifty-four channels (24 light sources and 16 detectors) mainly covered the frontal, temporal, and occipital lobes, which were determined according to the international 10-20 system (**Figure 3**). All participants will be instructed to sit quietly for 8 minutes. If they are restless, a research assistant will try to comfort them. Three types of caps (extra small, small, and medium) will be prepared for them.

### Data collection and management

Upon enrolment, the names of participants will be replaced with a four-letter code in order to safeguard their privacy. The data will be recorded in the case report form, which encompasses the following information: demographics, patient history, baseline, post-treatment evaluation, and record of adverse events. Following the completion of each phase of data collection, the quantitative data will be entered into a Microsoft Excel spreadsheet. The data collection process will be conducted by an independent assessor who will be unaware of the participant allocation. The entered information will be protected by a password that will only be accessible to members of the research team. Any personal information provided by trial participants will be treated in strict confidence. All data will be stored securely for at least five years after completing the study.

### Data analysis plan

All statistical analyses will be performed using IBM SPSS 26.0 by independent blinded statisticians. Chi-squared tests and t-tests will be performed for the categorical and continuous variables, respectively, to examine the group differences in the demographics and clinical profiles between the two groups at baseline. To assess for significant differences in treatment effects over time, a mixed-effects model with predictor variables for group (rTMS condition: active vs placebo) and time (baseline vs. end of treatment vs. follow-up) will be fitted to the data from the outcome measures. The effect size between groups will also be calculated. The feasibility and safety outcomes will be presented as proportions, and we will compare the rates between the two groups. All significance levels will be set at P < 0.05.

The EEG signals are preprocessed using the EEGLAB toolbox under MATLAB R2022b. A finite impulse response filter is constructed with a lower edge frequency of 1 Hz and a higher boundary frequency of 45 Hz. In order to artifacts induced by excessive body movements, we will perform an initial visual inspection of the EEG data. An independent component analysis will be conducted to remove additional artifacts. The segmentation will divide the EEG signals into epochs of 2s. The absolute and total power of the four frequency bands (δ, θ, α, and β) are calculated separately using the fast Fourier transforms. Then, the relative power of channels in these frequency bands will be calculated using MATLAB R2022b to analyze the changes in brain functional network characteristics before and after the intervention, including the magnitude squared coherence, phase locking value, phase lag index, and weighted phase lag index.

The NirSpark software package (Danyang HuiChuang Medical Equipment Co., Ltd., Zhenjiang, China) was used to analyze the fNIRS data. Following the conversion of the raw data to optical density signals, the motion artifacts will be reduced through the utilization of cubic spline interpolation. The obtained signals are filtered with a band-pass (0.01 to 0.2 Hz) filter in accordance with Butter’s law, to eliminate physiological noise generated by heartbeat and respiration. After preprocessing, the optical density will be converted to concentrations of oxyhemoglobin, deoxyhemoglobin, and total hemoglobin based on the Beer-Lambert law.

### Data monitoring

This study does not have a coordinating center or trial steering committee because it is single-center. This study does not require the appointment of a Data Monitoring Committee due to the absence of interim analyses. The Ethics Committee of the China Rehabilitation Research Center will oversee the study, including monitoring for adverse events, potential protocol bias, and study progress. Access to the study results will be granted to the principal investigator.

### Safety monitoring

Clinicians will monitor and record adverse events from the time informed consent is obtained until the final study visit. A medical and medication history questioning and an EEG will be carried out prior to randomization, and if any safety issues are identified (e.g. potential risk of epilepsy), the participant will be excluded. Even so, we will prepare a finger-clip oximeter, oxygen bags, tongue depressors, and antiepileptic medication to address the potential occurrence of an epileptic seizure, which represents one of the most serious adverse effects associated with rTMS. Parents will be encouraged to report any new or worsening symptoms of their children, which will be evaluated for possibilities with the iTBS intervention.

### Patient and public involvement

This clinical protocol was reviewed by the Ethical Committee of the China Rehabilitation Research Center, parents of ASD children, and public advocates. We have modified the protocol accordingly.

### Ethics and dissemination

The Ethics Committee of the China Rehabilitation Research Center approved this study. Any significant change to the protocol must be approved by the Office of Academic Research and the Ethics Committee at the China Rehabilitation Research Centre, according to the protocol amendment application procedures. The protocol amendment will also be reported to the China Clinical Trial Centre. All legal guardians of participants will be provided with detailed information regarding the study and will consent to participation before assessments. The results of this study will be disseminated via publishing in an international peer-reviewed journal and will be made available to participants upon request.

## Discussion

To our knowledge, this study is the first to investigate the feasibility, safety, and efficacy of high-dose iTBS in children with ASD. We will assess recruitment, treatment adherence, tolerability, and safety of this modified rTMS protocol in the field of ASD. We will also examine its preliminary efficacy and provide novel information on brain function changes that occur during iTBS treatment. The insights gained from salivary oxytocin and growth-related factor assessments will contribute to a more profound comprehension of the role of iTBS in influencing the hypothalamic-pituitary system of ASD. If successful, larger-scale studies will be needed to ascertain the intervention’s efficacy with greater precision.

Several limitations should be noted. The study is currently being conducted on a feasibility scale and includes a small size which may be insufficient to detect significant differences between two groups. Relying solely on recruiting patients from the hospital may limit the generalizability of our findings. We decided to include children with ASD regardless of gender, subtype, and severity, leading to a heterogeneous population that may influence the study results. Last but not least, the lack of neuroimaging-guided target localization is a limitation of our iTBS program.

In conclusion, this trial will evaluate the feasibility, safety, and efficacy of high-dose iTBS treatment in children with ASD. The proposed study will provide pilot data to inform the feasibility and design of larger sample-size trials.
